# Verification of mathematical models of response threshold through statistical characterisation of the foraging activity in ant societies

**DOI:** 10.1038/s41598-019-45367-w

**Published:** 2019-06-20

**Authors:** Osamu Yamanaka, Masashi Shiraishi, Akinori Awazu, Hiraku Nishimori

**Affiliations:** Department of Mathematical and Life Sciences, Graduate School of Science, Hiroshima University, Higashihiroshima, Hiroshima 739-8526 Japan

**Keywords:** Behavioural ecology, Animal behaviour, Biological physics

## Abstract

The concept of response threshold (RT) has been developed to explain task allocation in social insect colonies, wherein individual workers engage in tasks depending on their responsiveness to the task-related stimulus. Moreover, a mathematical model of RT has been proposed to explain data obtained from task allocation experiments; however, its applicability range warrants clarification through adequate quantitative analysis. Hence, we used an automatic measuring system to count passage events between a nest chamber and a foraging arena in five colonies of ants, *Camponotus japonicus*. The events were measured using radio-frequency identification tags attached to all workers of each colony. Here, we examined the detailed forms of i) labour distribution during foraging among workers in each colony and ii) the persistence of rank-order of foraging among workers. We found that labour distribution was characterized by a generalized gamma-distribution, indicating that only few workers carried out a large part of the workload. The rank-order of foraging activity among workers in each colony was maintained for a month and collapsed within a few months. We compared the obtained data with testable predictions of the RT model. The comparison indicated that proper evaluation of the mathematical model is required based on the obtained data.

## Introduction

The mechanism underlying flexible organization of complex tasks allocated to various workers in colonies of social insects has been widely explored^1,2^. To conceptually describe the task allocation mechanism in a colony of honeybees, Robinson proposed a response threshold concept (RT concept)^1,3^. This concept assumes that i) the responsiveness of workers to stimuli varies with time and depends on endogenous levels of a juvenile hormone in the workers, indicating that the primary engaging task is regulated by response thresholds^3–7^ and ii) the environmental and colony conditions determine the probability of engaging tasks for the workers. The response thresholds of workers are determined by various factors including genetic variation^8,9^, experience^10,11^, body size^12–15^, age^16–18^ and spatial distribution of each task^19^. Thus far, the availability of the RT concept to describe the division of labour among workers has been assessed primarily on the basis of i) difference in responsiveness to stimuli among workers belonging to different castes^3–5,7–9,12,20–25^ and ii) time-variation in responsiveness^3,16–18,26^.

To describe the RT concept mathematically, Bonabeau *et al*. proposed the fixed response threshold (FRT) model^27^. The FRT model assumes that a) each worker has a set of task-dependent thresholds to start the corresponding set of tasks and that b) stresses, which represent the demands for respective tasks in a colony, are shared by all workers in a colony. According to the FRT model, a worker with a lower threshold for a task has a higher probability of performing the task than those with higher thresholds. Consequently, the stress level of the colony for each task decreases as more workers with lower response thresholds engage in the corresponding task (formulae are provided in Supplementary Note 1). In addition, the FRT model assumes that workers in a caste have a caste-specific low response threshold for a specific category of tasks. This means that the workers of a specific caste have a higher preference for performing the corresponding task than workers outside the caste.

In addition to describing the task allocation, the FRT model has been considered to explain labour distribution (workload distribution) among workers in a colony^27^. On the labour distribution, two hypotheses are deduced from the mathematical character of the FRT model. First, considering that the FRT model is inherently a stochastic model and that in this model the same probability of performing each task is shared by all workers within each caste, variation in the workload among them should arise only from statistical fluctuation. Therefore, if the FRT model holds true, the distribution of the workload among the workers in the same caste converges to a single peaked normal distribution. We call this hypothesis on the convergence of the workload distribution (H1) the equally distributed workload hypothesis among workers. This hypothesis works even if the workers are in charge of multiple tasks. The workload distribution among a group of workers belonging to more than one but a finite number of different castes should show a superposition of normal distribution. Our second hypothesis states that the rank-order of task activity is replaced randomly. Thus, the characteristic replacement period is very short. Like (H1), this hypothesis is directly deduced from the stochastic dynamics defined in the FRT model. We call this hypothesis on the rank-order replacement (H2) the sustainable and rapid replacement hypothesis of rank-order among workers. The main issue of the present study is to evaluate whether these hypotheses reflect reality, by analyzing a sufficient amount of behavioural data of the foraging task.

The FRT model, which was originally proposed to describe the compensability of already-organized task allocation structures in ant colonies, was later improved to explain the emerging process of task allocation by Theraulaz *et al*.^28^. In this improved model called response threshold reinforcement (RTR) model, RTs of individual workers are updated with time through feedback; in brief, if a worker occasionally meets the demand for engaging in a task, the corresponding RT of the worker decreases. Because of this decrease, the opportunity for the worker to start engaging in the same task at the subsequent opportunity increases (formulae are provided in Supplementary Note 2). Furthermore, we could revise the homogenious positive feedback rule among workers to construct much complicated form of the RTR model. This model would allow a wider variety in the workload distribution than that expected from the FRT model.

In spite of their high potentiality to describe various aspects of phenomena, we consider that basic assumptions of the family of RT models (i.e., the FRT model and the RTR models) lack complete experimental confirmation at least for the labour statistics of ants. Thus, in this study, we focused on meticulously examining the quantitative aspects of the FRT model. For this purpose, we analysed a large set of behavioural data of individual ants, *Camponotus japonicus*, from five colonies (Colony A, B and C are queen-right, Colony D and E are queen-less). The data were obtained using radio-frequency identification (RFID) tags (SK-Electronics Co., Ltd.). We initially estimated the cumulative distribution of foraging activity fractions for each day. Thereafter, we analyzed the time course of the rank-order of foraging activity (ROoFA) of the five colonies. As subsequently indicated, upon continuous observation for several months, new characteristic features of the cumulative distribution and ROoFA, which were expected to provide a basis for quantitative estimation and data-supported improvement of the RT model.

## Results

**Figure 1:**
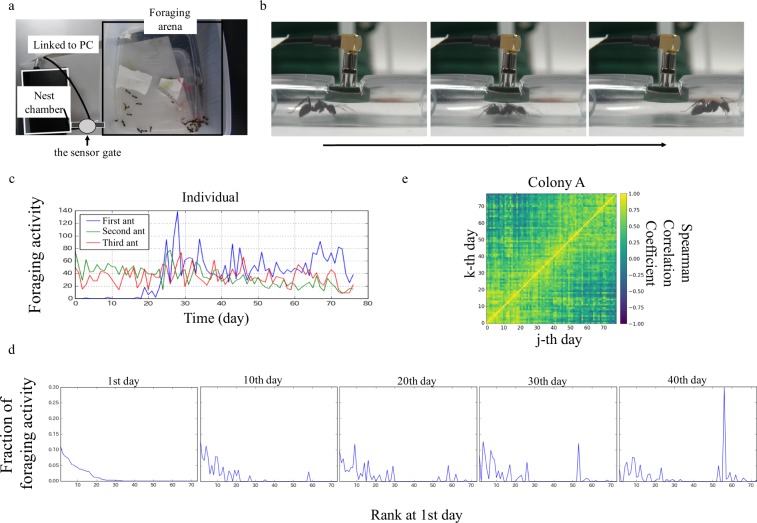
Automatic system for measuring the foraging activity of *Camponotus japonicus* colonies, using tiny radio-frequency identification (RFID) tags (SK-Electronics CO., Ltd.) (**a**) Top view of the experimental setup. (**b**) Side view of the sensor gate and snapshots of a passage event of an ant moving from the nest chamber (left) to the foraging arena (right) in a time sequence. (**c**) The time series of the number of passage events in each day of three of the most active ants in the same colony A. (**d**) The rank-order of foraging activity plots of 1st day and the rank foraging activity plots of typical days (10th, 20th, 30th, and 40th days) in ascending rank-order of foraging activity from day 1. (**e**) Spearman’s correlation coefficient (SCC) matrix of colony A determined from ROoFAs of every 2 d among workers who passes the sensor gate at least once in both days. The colour labels show Spearman correlation coefficient.

In total, 172,568 passage events of 750 workers from five colonies were recorded for approximately three months on average. The number of passage events of each worker on each day was termed the ‘daily foraging activity’ of the worker. The foraging activity, normalized by the total number of passage events among all workers in a colony on each day, was called the ‘daily foraging activity fraction’. The daily foraging activity of individual workers fluctuated with time (Fig. 1c). Furthermore, the daily foraging activity was skewed toward a small number of high-ranked workers, and the rank-order of daily foraging activity fluctuated with time (Fig. 1d).

### Cumulative distribution of daily foraging activity fractions

**Figure 2:**
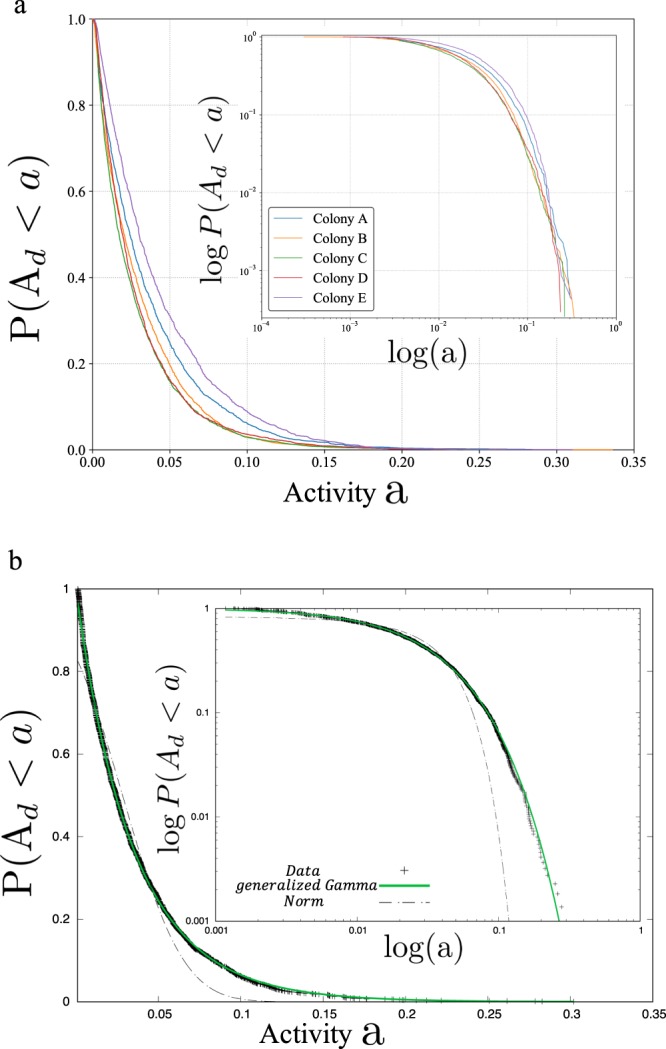
(**a**) Complementary cumulative fractions of daily foraging activities of colonies. (**b**) Complementary cumulative fractions of daily foraging activities of Colony A and the candidate curves for the cumulative form of the distribution function of Colony A.

The cumulative form of the daily foraging activity fractions defined by equation (2), did not fit the normal distribution nor the superposition of a finite number of normal distributions. This results indicates that the daily foraging activities were not just randomly distributed around the average (Fig. 2 and Supplementary Fig. S1). More specifically, the generalized gamma-distribution was found to best fit the cumulative distribution of daily foraging activity fractions based on the Akaike information criterion (AIC) (Table 1 and Supplementary Table S1).

**Table 1:** Table shows *α*, *β*, and *γ* of the fitting parameters and the Akaike information criterion values used to fit distributions to the cumulative fraction of daily foraging activities among ants in Colony A.

Colony	Distribution	*α* Estimate	Std.	*β* Estimate	Std.	*γ* Estimate	Std.	AIC
Colony A	*P*_*Gamma*_	0.964902	0.001898	0.036880	0.000095	—	—	−9169.174599
	*P*_*Weib*_	0.976743	0.001185	0.035324	0.000027	—	—	−9206.678831
	*P*_*gGamam*_	1.134471	0.019350	0.033972	0.000226	0.904186	0.009372	−9260.501741
	*P*_*Singh−Maddala*_	0.983762	0.003106	2.393440	1.023754	63.856774	25.858800	−9210.820508
	*P*_*Exp*_	—	—	28.350285	0.024188	—	—	−8872.456517
	*P*_*Norm*_	0.028326	0.000152	0.029062	0.000271	—	—	−3868.480563
	*P*_*log−Norm*_	−3.787273	0.002701	1.145896	0.004417	—	—	−6090.474977

### The time courses of the rank-order of foraging activity

To quantitatively investigate the time variation in the distribution of daily foraging activity within each colony, we focused on the time course of the ROoFA among workers in each colony. We calculated the Spearman correlation coefficient (SCC) of ROoFAs between arbitrary pairs of days. The SCC indicates the degree of the replacement of ROoFAs between a pair of days, i.e. if the ROoFAs between a pair of days remain the same, the SCC is nearly equal to 1. In contrast, if the ROoFAs between a pair of days are completely shuffled (i.e. a random replacement of the ROoFAs), the SCC is nearly equal to 0. The SCC matrix for an exemplary colony is shown in(Fig. 1e). Values for the diagonal elements of the matrix are 1 from the definition of the SCC. They tend to decrease with the distance from the diagonal region.

**Figure 3:**
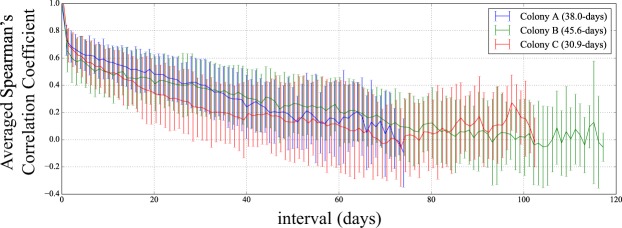
Spearman’s correlation coefficient averaged over all pairs of daily ROoFAs for respective day-differences corresponding colonies.

The averaged SCC between two successive days was positive and close to 0.7 (Fig. 3 and Supplementary Fig. S2). The estimated relaxation times of the SCC, namely the persistence times of the ROoFAs, were approximately one month for Colony A, Colony B and Colony C with 38 days, 45 days, 30 days, respectively. Colony D and Colony E showed longer relaxation times, 65 days and 100 days, respectively.

## Discussion

Despite the popularity of the FRT model in the study of social insects, little quantitative validation through experiment exists. have not sufficiently been conducted. Here, we aimed to evaluate the applicable range of FRT model by applying it to ant colonies. Based on previous applications of the model, we derived two hypotheses: (H1) the equally distributed workload hypothesis and (H2) the sustainable and rapid replacement of rank-order hypothesis. We tested our hypothesis by measuring the foraging activity of ants. Our tests yielded two relevant statistics: the cumulative distribution of foraging activity fraction and the ROoFAs. To compare the experimental results with our hypotheses, a sufficient amount of data is required for a statistically significant estimate of the cumulative distribution of foraging activity fraction and the correlation of the ROoFAs between different days. To obtain the necessary data, we introduced an automatic system to measure long-term foraging activity of ants using tiny RFID tags. We applied this system to observe the five colonies, and to store time series on passage events that workers walked through a narrow gate sensor between the nest chamber and the foraging arena with the identification of individual workers. The amount of data analysed in our study was much larger than in previous studies^29,30^.

As stated above, our analysis of the behavioural data obtained, yielded two main results. (R1) First, the cumulative distribution of foraging activity fractions was characterized via a generalized gamma-distribution. (R2) Second, time-course analysis of ROoFAs revealed that over the long term, ROoFAs were dynamic, with a persistence time of two months or longer. The first result (R1) means that our data were not consistent with the “equally distributed workload hypothesis” of the FRT model^27^. Note that the present data may have included not only genuine foraging activity data but also other types of behavioural data such as scouting. Even if this was the case, based on the concept of the stochastic FRT model, the workload distribution among workers belonging to more than one but a finite number of castes should fit a superposition of normal distributions, which was not the case of our data analysis. It indicates that our results on the cumulative distribution of workload are not described by the FRT model, irrespective of the number of castes.

To obtain the second result (R2), we compared the ROoFAs between pairs of days. We confirmed the strong persistence of the ROoFAs between successive days (i.e. S*CC*≃0.7). According to the FRT model, workers in a caste share almost identical RTs for a task, and the workload for the task undertaken by workers in a certain period is exclusively determined by stochasticity; hence, the rank-order of task activity among workers in the same caste should rapidly change (the sustainable and rapid replacement of rank-order hypothesis). In this regime our findings are inconsistent with the FRT model.

In addition to the two results discussed above, a systematic decrease in the time variation of SCC was identified, and its finite relaxation time was estimated to be one month or longer in both queen-right and queen-less colonies (i.e. 38 days, 45 days, 30 days, 65 days and 100 days for the respective colonies). This finding indicates a gradual but large shift of the workload among workers within a few months. The final saturation values show a difference between queen-right and queen-less colonies (Fig. 3 and Supplementary Fig. S2), which should be evaluated more comprehensively.

In conclusion, the original idea of the FRT model does not explain the present experimentally obtained outcomes. Indeed, the unequal distribution of workload among workers might be reproduced by assuming variation in RTs within the same caste. Time variation in the rank-order of a task activity among workers might be described by implementing complex feedback from interactions among workers and the colony condition into response thresholds. More in-depth discussions based on careful analyses of sufficient behavioural data are required to quantify the range of applicability of various versions of the RT model and understand the overall characteristics of collective behaviour of ants.

## Methods

### Material and Animal care

Five colonies of *Camponotus japonicus* were collected from the Higashihiroshima campus of Hiroshima University in June 2015 and June 2018. This species of ant is monogynous and polymorphic (the body size of individual workers in each colony is continuously distributed in the range of 7–12 mm)^31^. Three colonies, Colony A, Colony B, and Colony C contained one queen in each colony, and Colony D and Colony E did not contain any queen. Approximately 150 workers existed in all colonies. The colonies were maintained in plastic cases (sized 150 mm × 81 mm × 24 mm) patched with plaster to maintain humidity and wrapped in setting tape to prevent exposure to light. All walls in the foraging arena (242 mm × 306 mm × 103 mm) were coated with fullon to prevent workers from escaping; the foraging arena and nest chamber were connected by a rubber tube (Fig. 1a,b). In the experiment room, LED lights were turned on every day at 8:00 AM and turned off at 8:00 PM, the temperature was maintained at 25 °C, and the humidity was set above 50%. Mealworms were supplied once every two days in the foraging arena, and insect jelly was continuously supplied to maintain the foraging activity of workers. Experiments were conducted from May 1, 2015 to August 18, 2015, for Colony A, from June 3, 2018, to October 1, 2018, for Colony B, from June 6, 2018, to October 1, 2018, for Colony C, and from June 26, 2018, to October 1, 2018, for Colony D and Colony E.

### Tag attachment and measuring system

RFID tags with unique identification numbers were attached to the thoraxes of all workers, except for queen ants, using acrylic resin glue (Kiyohara UVR) without exposure to CO2. The weight of each RFID tag was less than 0.11 mg, its size was 0.5 mm × 0.5 mm × 0.05 mm, and the weight ratio of the ant to RFID tag was less than 0.1%. The weight of the RFID tags was lower than the weight of tags used in previous studies^32–35^; therefore, the effect of tags on worker behaviour was believed to be ignorable. After the RFID tags were attached to 10 workers, these workers were placed in a case and irradiated with ultraviolet light for 30 min to harden the glue. This process was repeated for all workers released in the foraging arena, before they returned to their nest chamber.

After the workers had been equipped with the tags, it took them several days to get used to the new nest chamber and foraging arena. To take this adjustment period into account, experiments started one week after the workers had entered the nest chamber. Newly emerged workers were not equipped with RFID tags to avoid the inclusion of data from post-onset workers. An RFID reader (sensor; herein referred to as the ‘sensor gate’) was attached to the ceiling at the midpoint of the narrow rubber tube (inner diameter) that connected the foraging arena and the nest chamber (Fig. 1a,b). The RFIDs of individual workers and corresponding time stamps were automatically relayed to a computer as workers passed under the RFID reader. The error rate in sensing passage events at the sensor gate was not zero (the error rate was 15% on average, according to a 90-minute trial, during which we checked video-recorded passage events of workers by eye and compared these results to automatically counted passage events using the RFID tag). Furthermore, the passage direction, from the nest chamber to the foraging arena or the reverse, was not distinguishable by this system.

### Analysis of foraging behaviour

Daily foraging activity was defined as the number of passage events of each worker through the gate sensor per unit day. Here, one day-unit was a 24-h period from the lighting time of a day to the same time the next day. Note that a ‘passage event’ does not necessarily indicate foraging behaviour and could, for example, indicate wandering behaviour among workers between two boxes. We believe, however, that this frequency of passage events is an efficient index to estimate the degree of foraging activity, because the sensor gate was located at a unique passage connecting the foraging arena and the nest chamber^32^.

### Cumulative distribution of the daily foraging activity fraction

We defined *A*_*d*_ as the daily foraging activity fraction, which was represented by the following expression:

1 $${A}_{d}(i,m)=\frac{{d}_{i}(m)}{{\sum }_{j}^{{N}_{m}}{d}_{j}(m)},$$

where *d*_*j*_(*τ*) is the above mentioned daily foraging activity of the j-th worker on the *τ*-th day, and *N*_*τ*_ is the total number of workers that passed through the sensor gate at least once on the *τ*-th day. Here, we estimated the cumulative form of the daily foraging activity fraction, which was defined as follows:

2 $$P({A}_{d} < a)=\frac{\#\{(i,m)\,0 < {A}_{d}(i,m) < a\}}{\#\{(i,m);0 < {A}_{d}(i,m)\le 1\}},$$

where 0 < *a* ≤ 1. This quantity characterized the allocation of foraging workloads among workers on each day. We explored the best fit function for the cumulative fraction of *A*_*d*_ averaged over the experimental period. Hence, we prepared various candidates for the cumulative form of the distribution function, namely, gamma, Weibull, generalized gamma, Singh-Maddaia, exponential, normal, and log-normal distributions. The specific forms of these distribution functions are shown in Table 2 We used AIC to determine the best fit.

**Table 2:** Candidates for the cumulative distribution of daily foraging activity fractions. *α*, *β* and *γ* are parameters related to data distribution, Γ(*k*) is the gamma function, Γ(*s*, *x*) is the incomplete gamma function, and erf is the error function.

	Notation	Form of cummulative distribution
Gamma distribution	*P*_Gamma_(*X* < *a*)	$\frac{{\rm{\Gamma }}(\alpha ,\beta a)}{{\rm{\Gamma }}(\alpha )}$
Weibull distribution	*P*_*Weib*_(*X* < *a*)	$1-\exp (-{(\frac{a}{\beta })}^{\alpha })$
Generalized gamma distribution	*P*_*gGamam*_(*X* < *a*)	$\frac{{\rm{\Gamma }}(\alpha ,{(\frac{a\alpha }{\beta })}^{\gamma })}{{\rm{\Gamma }}(\alpha )}$
Singh-Maddaia distribution	*P*_*Singh*−*Maddala*_(*X* < *a*)	$1-{(1+({(\frac{a}{\beta })}^{\alpha })}^{-\gamma }$
Exponential distribution	*P*_*Exp*_(*X* < *a*)	1 − exp(−*βa*)
Normal distribution	*P*_*Norm*_(*X* < *a*)	$\frac{1}{2}(1+{\rm{erf}}(\frac{a-\alpha }{\sqrt{2{\beta }^{2}}}))$
log-Normal distribution	*P*_*log*−*Norm*_(*X* < *a*)	$\frac{1}{2}(1-{\rm{erf}}(\frac{\mathrm{ln}\,a-\alpha }{\sqrt{2{\beta }^{2}}}))$

### The time courses of the rank foraging activity

To determine the rank foraging activity, we defined the daily ROoFA. The correlations between arbitrary pairs of the daily ROoFAs obtained were quantified as follows:

3 $${M}_{j,k}=Cor{r}_{S}(r({S}_{j,k})),{S}_{j,k}=\{({d}_{i}(j),{d}_{i}(k)):0 < {d}_{i}(j),0 < {d}_{i}(k),\,\forall \,i\},$$

where *i* is the index of individual workers with attached RFIDs throughout the experimental period, and *j* and *k* are day-indicators, where *S*_*j*,*k*_ is the activity list of workers who passed through the sensor gate at least once on both the i-th and k-th days. *r*(*S*_*j*,*k*_) represents the function of transforming activity lists *S*_*j*,*k*_ of workers on the i-th and k-th days into rank-orders of foraging activity on respective days. *Corr*_*S*_(*r*(*S*_*j*,*k*_)) represents the SCC between rank-orders of foraging activity on the i-th and k-th days. Here, we calculated the averaged SCC as follows: $Corr(D)=\frac{1}{\#D}{\sum }_{|j-k|=D,|{S}_{j,k}| > 3}{M}_{j,k}$ where #*D* is the number of pairs of days that satisfy |*j* − *k*| = *D*, and |*S*_*j*,*k*_| is the number of elements in the list *S*_*j*,*k*_. By determining *Corr*(*D*), we estimated the persistence period of the ROoFAs, namely, the characteristic relaxation time of the averaged SCC as a function of the intervals (day-difference) between pairs of days. Note also that we considered data only from workers who passed through the gate sensor at least once on both of the *j*-th and the *k*-th days.

### Statistics

We used the Levenberg-Marquardt method for fitting the workload distribution with the candidate distribution and for fitting *Corr*(*D*) with the exponential curve. This computation was accomplished using the R software (version 3.3.1) with packages minpack.lm^36,37^.

## Supplementary information

Supplementary information
