# Supplementary material for: Verification of mathematical models of response threshold through statistical characterisation of the foraging activity in ant societies
Source: Sci Rep. 2019 Jun 20;9:8845. doi: 10.1038/s41598-019-45367-w (PMC6586672; doi:10.1038/s41598-019-45367-w)
Supplement: Supplementary file 1 — Supplementary information [file 41598_2019_45367_MOESM1_ESM.pdf]

Supplementary information for:
Verification of mathematical models of response threshold through statistical characterisation of the foraging activity in ant societies

Author: Osamu Yamanaka[1],Masashi Shiraishi[1],Akinori Awazu[1],Hiraku Nishimori[1]

S1: Supplementary Note S1

S2: Supplementary Note S2

S3: Supplementary Figure S1

S4: Supplementary Figure S2

S5: Supplementary Table S1

S1: Supplementary Note S1

Fixed response threshold model The fixed response threshold model is described by P_i and $s(t)$. P_i is the probability that an ant engages or disengages in a task in unit time and is a function of $s(t)$ and θ_i which is defined as the following equations:

$$P_i(\text{inactive} \to \text{active}; t) = \frac{s^2(t)}{s^2(t) + \theta_i^2} \quad (1)$$

$$P_i(\text{active} \to \text{inactive}; t) = p \quad (2)$$

where θ_i is the RT of an ant of an index i, $s(t)$ is the stress level as defined below and p is constant value, which determines the probability an ant gives up performing a task in unit time. $s(t)$ is defined as follows:

$$s(t+1) = s(t) + \delta - \alpha \frac{N_{\text{active}}}{N} \quad (3)$$

where N_{active} is the number of ants engaging at a time t, N is the number of ants in a colony, and α is a scale factor, which determines the workload carried out by an ant in unit time.

S2: Supplementary Note S2

Response threshold reinforcement model In the response threshold reinforcement model, the time variation of RTs is described as following equation:

$$\theta_i = \begin{cases} \theta_i - \xi & \text{(if i-th ant is active)} \\ \theta_i + \phi & \text{(if i-th ant is inactive)} \end{cases} \quad (4)$$

where ξ and ϕ are the variability rate of sensitivity to tasks.

S3: Supplementary Figure S1

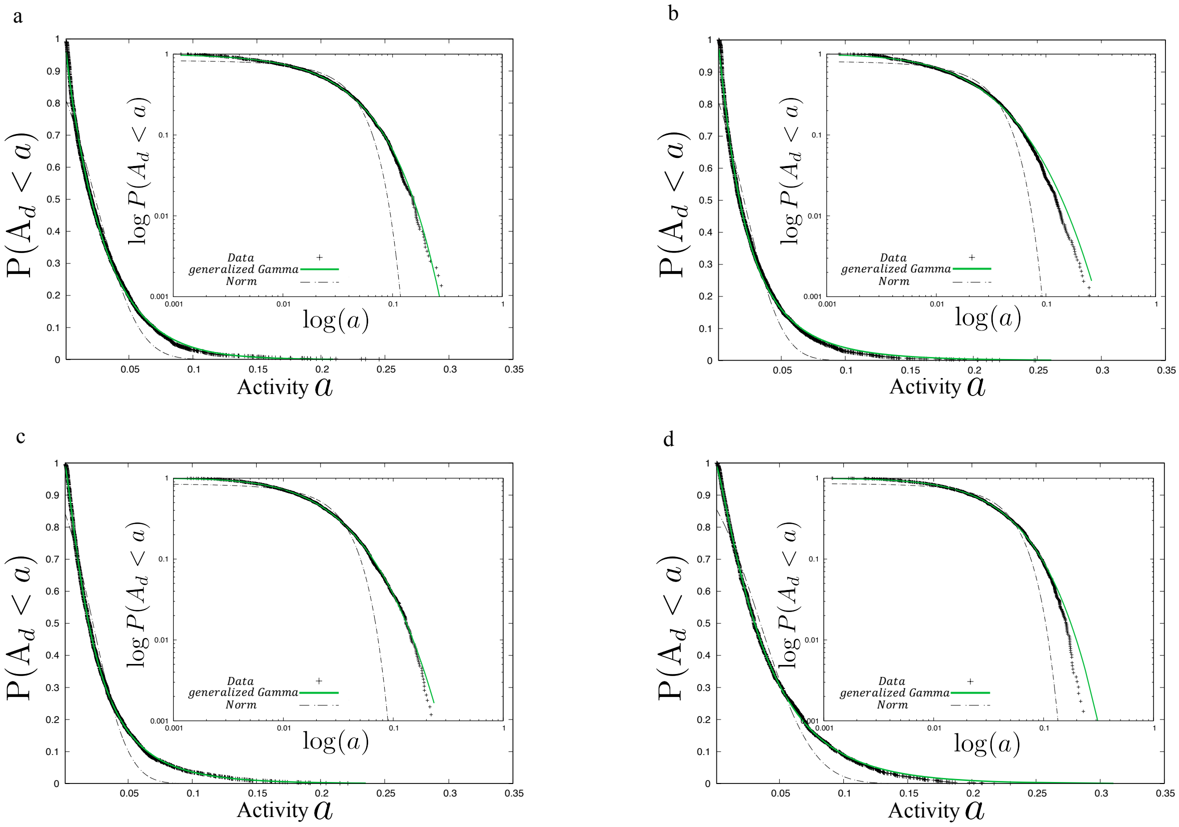

Figure S1. Complementary cumulative fractions of daily foraging activities of each colony and the candidate curves for the cumulative form of the distribution function of corresponding colonies(a : Colony B, b : ColonyC, c : Colony D, d : Colony E).

S3: Supplementary Figure S2

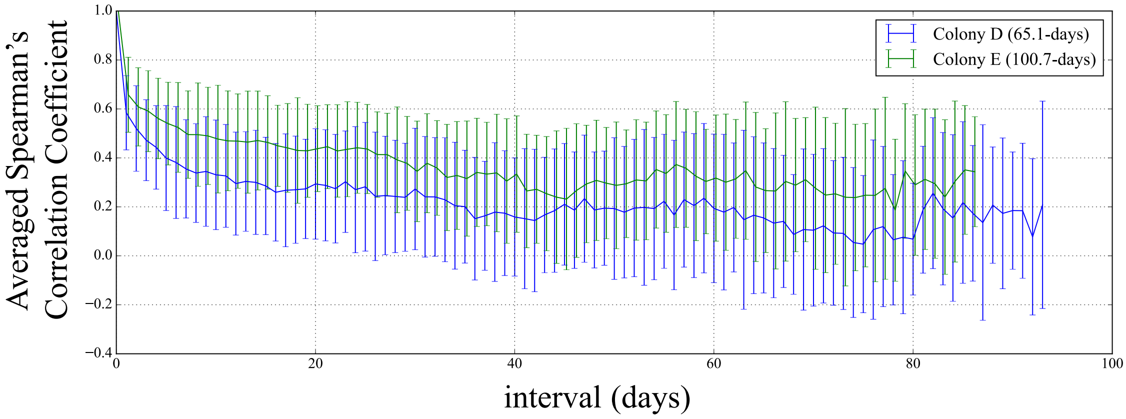

Figure S2. Spearman's correlation coefficient averaged over all pairs of daily ROoFAs for respective day-differences of corresponding colonies.

S5 Supplementary Table

Table S1. α, β, and γ of the fitting parameters and the Akaike information criterion values used to fit distributions to the cumulative fraction of daily foraging activities for Colony B, C, D and E.

Colony	Distribution	α		β		γ		AIC
		Estimate	Std.	Estimate	Std.	Estimate	Std.	
Colony B	P_{Gamma}	0.938352	0.002315	0.032377	0.000104	-	-	-9959.431002
	P_{Weib}	0.962378	0.001490	0.029970	0.000029	-	-	-9923.732626
	P_{gGamam}	0.845988	0.016795	0.030754	0.000048	1.067790	0.013434	-9981.564296
	$P_{Singh-Maddala}$	0.962418	0.001509	515.834383	3938.275026	11930.941681	87771.758571	-9921.434437
	P_{Exp}	-	-	33.399117	0.037203	-	-	-9393.683175
	P_{Norm}	0.023884	0.000115	-0.026359	0.000214	-	-	-4864.057164
	$P_{log-Norm}$	-3.955895	0.002814	1.150842	0.004567	-	-	-6881.532631
Colony C	P_{Gamma}	0.962765	0.004105	0.027558	0.000150	-	-	-7759.534784
	P_{Weib}	0.969390	0.002474	0.026322	0.000042	-	-	-7826.092131
	P_{gGamam}	3.729001	0.179740	0.004732	0.000624	0.466677	0.011788	-8835.865994
	$P_{Singh-Maddala}$	1.112189	0.006663	0.068370	0.003431	3.451470	0.145507	-8337.789216
	P_{Exp}	-	-	38.010072	0.062358	-	-	-7682.320170
	P_{Norm}	0.020917	0.000128	-0.023122	0.000237	-	-	-4007.961713
	$P_{log-Norm}$	-4.080758	0.001850	1.122569	0.002875	-	-	-7719.437584
Colony D	P_{Gamma}	1.135539	0.003955	0.024158	0.000104	-	-	-7907.028718
	P_{Weib}	1.071189	0.002485	0.028098	0.000038	-	-	-7642.036317
	P_{gGamam}	4.641454	0.112045	0.003659	0.000268	0.456972	0.005744	-10432.288651
	$P_{Singh-Maddala}$	1.284550	0.004342	0.047319	0.000922	2.500079	0.045578	-9396.548819
	P_{Exp}	-	-	35.478780	0.064022	-	-	-6960.898665
	P_{Norm}	0.022604	0.000121	-0.021620	0.000213	-	-	-3830.243681
	$P_{log-Norm}$	-3.975594	0.001286	1.026643	0.002014	-	-	-8013.994765
Colony E	P_{Gamma}	1.176348	0.002631	0.035945	0.000100	-	-	-8071.116288
	P_{Weib}	1.099643	0.001759	0.043549	0.000040	-	-	-7674.004796
	P_{gGamam}	1.952518	0.035704	0.031011	0.000561	0.743815	0.007630	-8801.813598
	$P_{Singh-Maddala}$	1.197378	0.003927	0.161235	0.006136	5.363048	0.196259	-8286.889060
	P_{Exp}	-	-	22.817687	0.044410	-	-	-6020.977560
	P_{Norm}	0.035133	0.000178	-0.032321	0.000311	-	-	-3492.780108
	$P_{log-Norm}$	-3.528030	0.001981	1.009131	0.003195	-	-	-6017.544735
